# Physical fitness changes in adolescents due to social distancing during the coronavirus disease pandemic in Korea

**DOI:** 10.7717/peerj.14494

**Published:** 2022-12-16

**Authors:** Kwang-Jin Lee, Se-Young Seon, Byungjoo Noh, Keun-Ok An

**Affiliations:** 1 Department of Physical Education, Chungbuk National University, Cheongju, South Korea; 2 MSC Exercise Center, Seoul, South Korea; 3 Department of Kinesiology, Jeju National University, Jeju, South Korea; 4 Sports Medicine Major, Division of Sports, Korea National University of Transportation, Chungju, South Korea

**Keywords:** Physical activity, Physical fitness level, Middle school students

## Abstract

**Background:**

At least 60 min of moderate-intensity physical activity per day is recommended for physical and mental health of adolescents. Schools are one of the most suitable places for promoting students’ health as it is a place where vigorous physical activity occurs. However, the physical activity of students is threatened because schools are closed worldwide owing to the coronavirus disease (COVID-19) outbreak in 2019. Therefore, this study aimed to analyze the physical fitness changes in 27,782 Korean adolescents during the pandemic and present alternative education and health policies to the school.

**Methods:**

We included 29,882 middle school students (age: 13–15 years; males: 14,941, females: 12,841) in Korea from 2019 to 2021 . Participants’ physical fitness at school was measured using the physical activity promotion system (PAPS) manual developed to measure students’ physical fitness. Physical fitness variables included body mass index (BMI), 20 m shuttle run, handgrip strength, sit-and-reach, and 50 m run.

**Results:**

The COVID-19 pandemic has had a negative impact on the BMI and cardiorespiratory endurance of Korean middle school students. Specifically, male students’ BMI increased, while body composition, cardiorespiratory endurance, and grip strength decreased significantly. Female students showed significant decreases in BMI and sit-and-reach test scores. It is clear that the physical fitness level of adolescents decreased by a greater degree after the COVID-19 pandemic than before, and the decrease in the physical fitness level of male students was noticeable. Therefore, a lesson strategy should be prepared that considers the contents and methods of physical education classes to improve the physical fitness level of male and female adolescents.

**Conclusions:**

Fitness-based classes suitable for online methods should be urgently added as alternative physical education classes to prepare for the second COVID-19 outbreak. In addition, it is necessary to create an environment in which physical activity is a possibility in physical education classes, in any situation using artificial intelligence and virtual reality.

## Introduction

Physical activity (PA) refers to all the movements that use energy, skeletal and muscular strength, which has positive effects. Physical fitness has various physiological and psychological benefits and plays a vital role in bringing positive human health changes (Rhodes et al., 2017; Bell et al., 2019). It reduces mortality and prevents lifestyle diseases (diabetes, cancer, heart disease, and high blood pressure, etc.) (Rhodes et al., 2017). In addition, it improves psychological aspects that reduce depression, anxiety, and stress (Bell et al., 2019) and provides opportunities to form positive social relationships, such as improved peer relationships through cooperation and school life adaptation (Fitzgerald, Fitzgerald & Aherne, 2012). In particular, the amount of physical fitness during childhood and adolescence can affect population health. A high physical fitness level and different physical activities reduce the risk of health problems, such as obesity, cardiovascular disease, and mental disorder (Rangul et al., 2012; Masanovic et al., 2020). Therefore, to achieve optimal benefits, physical fitness in childhood must continue to improve into adulthood. A better understanding of adolescent physical fitness levels and PA is needed to establish a natural link between physical fitness and adulthood. A better understanding of adolescent physical fitness levels and PA is needed to establish a natural link between physical fitness and adulthood. The World Health Organization (WHO) recommends an average of 60 min of moderate or more (moderate to vigorous) PA (exercise) every day, considering its effects on the development, physical fitness and mental health, and overall life of adolescents (WHO, 2020a). Schools are well suited for promotion of physical fitness and PA among the youth. Young people spend 6–7 h a day at school for 40 weeks per year and engage in PA for approximately 40 min, four times a week, through attending regular physical education classes and after-school PA (Ministry of Education, 2022a; Ministry of Education, 2022b). Physical education classes in Korea have a significant impact on the physical activity level of adolescents, and that >51.4% of adolescents engaged in vigorous physical activity at least once a week during PE class (Yu et al., 2018; Lee & Yang, 2021). Schools have well-equipped facilities, qualified staff, and a curriculum for youth physical fitness (Lubans et al., 2016). Moreover, schools have the power to help achieve at least 60 min of moderate-intensity daily activity, as recommended by national or health groups (Gill et al., 2016).

The role of schools and communities in the physical fitness of students has been emphasized worldwide (McMahon et al., 2017). Therefore, the Korean Ministry of Education has revised the physical examination system completely. It has introduced a new student health and fitness evaluation system, known as the PA promotion system (PAPS) (Ministry of Education, 2009). Furthermore, the influence of health predictions in adulthood and social and environmental variables affecting students’ health can be analyzed.

In March 2020, the WHO declared coronavirus disease (COVID-19) to be a pandemic, thereby, alerting the entire world (WHO, 2020b). In response to COVID-19, authorities implemented a range of measures, including school closures, stricter quarantines, and recommended social distancing to mitigate the spread of the virus and reduce its burden on the healthcare system (Choi, 2020). In such a scenario, adolescents were exposed to weight gain, psychological anxiety, and decreased PA and physical fitness (Al Hourani, Alkhatib & Abdullah, 2021; Mittal, Firth & Kimhy, 2020). During this period, schools failed to provide moderate or high PA to students, thereby, losing their power as a public institution related to PA. Many countries investigated the physical fitness changes in adolescents during the lockdown period. They showed reduced agility, flexibility (Basterfield et al., 2022), lower (Tsoukos & Bogdanis, 2021) and upper extremity strength, and cardiopulmonary endurance (Wahl-Alexander & Camic, 2021). However, there is a limit to generalizing these results because the surveyed area and the number of participants was insufficient, and adolescents living in Asia were excluded from the previous study. Our research focused on these limitations, and the changes in physical fitness before and after COVID-19 were analyzed using panel data from middle school students throughout Korea. Therefore, our study analyzed the changes in physical fitness among Korean adolescents during the pandemic. In addition, based on the analyzed data, we also present an insight into post-COVID education and health policies in schools.

## Material and Methods

### Participants

**Figure 1:**
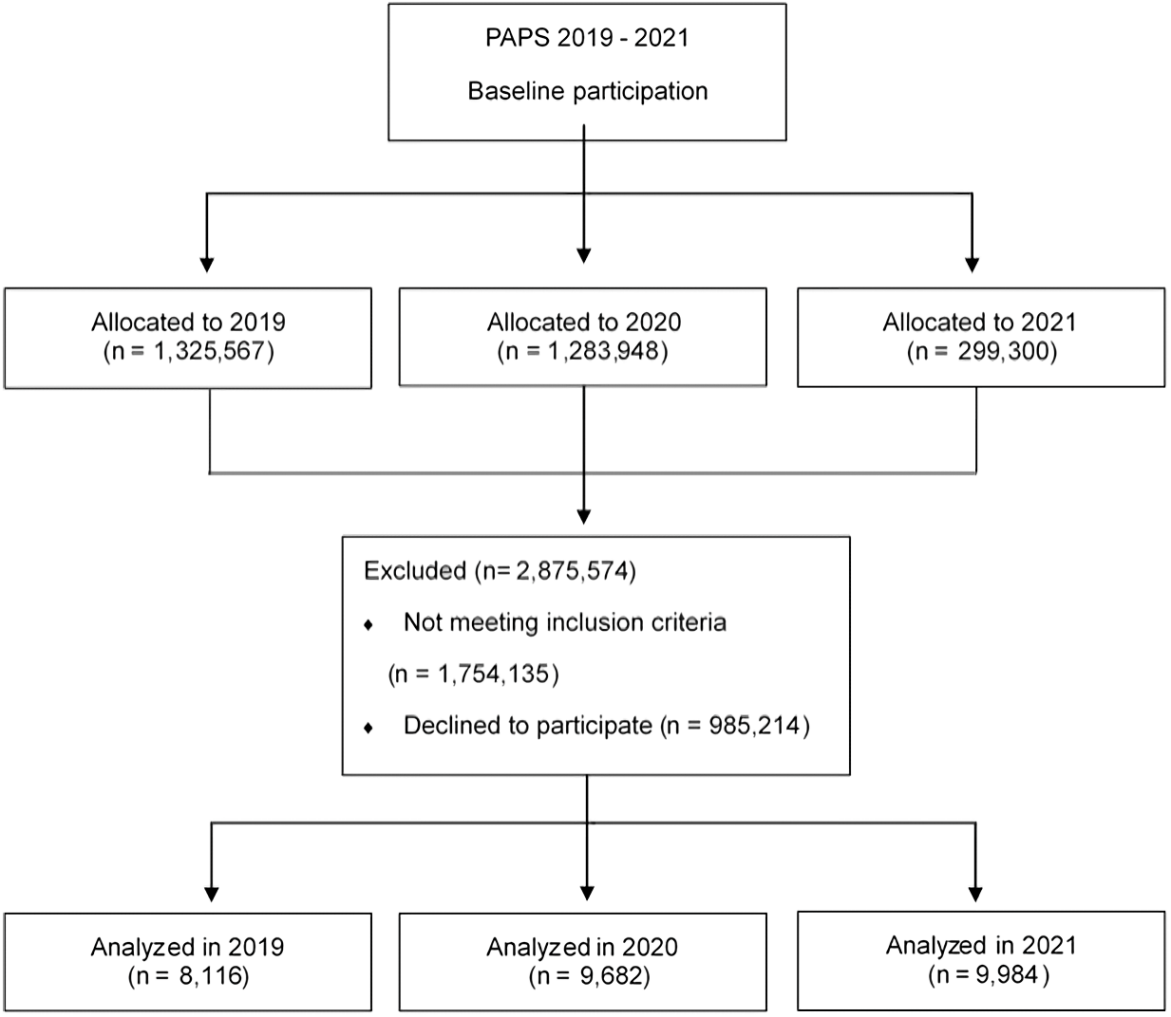
PAPS 2019–2021 participant flow diagram.

This study used public data (total number of participants, *n* = 27,782) from the Korean Ministry of Education. Data acquisition was performed by downloading the PAPS records of middle school students from 2019 to 2021, published on the site, school info (https://www.schoolinfo.go.kr/ng/go/pnnggo_a01_l2.do). The data were analyzed by selecting the same school and grade in 2019 and 2021, based on the school and grade used to measure the PAPS in 2020. COVID-19 was at a peak in 2020; thus, most schools in Korea were closed for 68 weeks (Ministry of Education, 2022a; Ministry of Education, 2022b). Among variables for physical fitness evaluation through PAPS, we included only those students who participated in the physical evaluation of body composition, shuttle run, grip strength, sit-and-reach, and 50 m run in the study. Consequently, 8,116 students in 2019, 9,682 students in 2020, and 9,984 middle school students in 2021 participated (Fig. 1). The exclusion criteria were as follows: (1) The data that do not include body composition, shuttle run, grip strength, sit-and-reach, and 50 m among the factors for physical fitness evaluation (Not meeting inclusion criteria, *n* = 1,754,135); (2) Those that refused to measure during the COVID-19 (Declined to participate, *n* = 985,214); (3) Those with changes in physical fitness measurement items between 2019 and 2021 (inconsistency of measurement variables, *n* = 136,225); (4) The data was fewer than 30 students measuring PAPS (Other reasons, *n* = 5, 459).

### Physical fitness test

The Ministry of Education developed the PAPS, and its guidelines distributed to schools and metropolitan/provincial offices of education so that PAPS can be measured in the same way as possible at school sites (see Table 1). The purpose of PAPS implementation is evaluation of students’ health and physical fitness to prevent deterioration of physical fitness, adult diseases, and obesity among students and to foster healthy students. In particular, all measurement results can be searched using Web-PAPS, and various PA prescriptions can be provided based on the evaluation results (Ministry of Education, 2009). Therefore, the physical fitness level of adolescents can be evaluated on a yearly basis using the PAPS. We assessed the following domains of physical fitness: body composition, cardiorespiratory endurance, muscle strength, flexibility, and power. The participants underwent the physical fitness test twice and the highest values of the results were recorded. The following shows the measurement items and methods for each factor in the student health and physical fitness evaluation (Ministry of Education, 2009). All measurements were directly performed by the physical education teacher of each school. Students were thoroughly instructed on the movements, and demonstrations were shown by the teachers before measurement. Measuring tools required for measurement may not be from the same manufacturer.

**Table 1:** Changes in PAPS of middle school students by year.

	2019^a^ (*n* = 8,116)	2020^b^ (*n* = 9,682)	2021^c^ (*n* = 9,984)	*X* ^2^	*p*-value	Post hoc
BMI (kg/m^2^)	21.5 ± 1.1	21.4 ± 1.0	22.0 ± 1.1	17.506	<0.001^***^	c >a, b
Shuttle run (rep)	46.4 ± 13.3	43.2 ± 14.5	37.8 ± 13.8	20.723	<0.001^***^	a, b >c
Grip strength (kg)	30.0 ± 65.7	28.5 ± 5.8	29.7 ± 6.9	2.884	0.236	–
Sit and reach (cm)	11.9 ± 3.7	12.0 ± 12.9	12.9 ± 3.7	3.697	0.158	–
50 m run (sec)	8.9 ± 0.9	9.0 ± 0.8	9.2 ± 1.0	5.670	0.059	–

**Notes.**

mean ± SD mean and standard deviation BMI body mass index rep. repetition

*** *p* < 0.001

Kruskal–Wallis test & Tukey test using ranks.

1. BMI (kg/m^2^) was calculated by dividing the weight by the square of the height. Height, weight, and BMI were measured using an automatic height/weight scale (GL-150, G-Tech International, Korea).
2. The 20 m shuttle run (time, second) was performed continuous running between 20 m lines to assess cardiorespiratory endurance (Leger & Gadoury, 1989). A marker cone was installed at a distance of 20 m. The subject was asked to start running with the starting signal, arrive at the opposite point before the following beep, and continue to perform the motion in accordance with the repeated beep. This informed the subject that the beep interval decreased stepwise. If the subject did not arrive at the opposite point before the beep sounds, he/she returned to his/her place again, and the evaluator marked “△” on the recording sheet If the second movement was not completed before the beep, the measurement was stopped. This rule was only applied once.
3. Handgrip strength (kg) was measured to assess muscle strength. The subjects raised their body in an upright position with both feet shoulder-width apart. The participants adjusted the width of the digital grip strength dynamometer (TKK-5401, Takei, Japan) to fit their hands. During the measurement, the subject was not supposed to bend the arm or move the upper body up and down.
4. Sit-and-reach (cm) was used to assess flexibility. The subject was supposed to touch the measuring instrument with the soles of their feet in a sitting position. The upper body was bent forward, and the arms were stretched forward. The posture was to be maintained with both arms extended at the same time and back recoil or bending of knees was not to be used. This number was recorded by maintaining the posture for more than 2 s with the upper body bent.
5. The 50 m run (sec) was performed to assess power. The subject was instructed to run forward at the fastest speed from the starting point to the 50 m arrival point in response to a whistle or flag signal. The subjects started in a standing position. When the evaluator was ready to measure and the subject was ready to perform, a command ”On Your Marks” was given. The start signal officer issued the command “Caution”, and after a suitable time (2–3 s), raised a flag to send a “start” signal to the subject. If a subject started or fell at a false signal, he/she was given time and measurement was taken after 20 min. The evaluator measured on a straight line, and the unit was recorded up to 0.01. Measured values were rounded up to the first decimal place.

### Statistical analysis

All statistical analyses were performed using SPSS (version 25.0; IBM Corp., Armonk, NY, USA). The distribution of normality in the data was confirmed using the Kolmogorov–Smirnov and Shapiro–Wilk tests. The Kruskal–Wallis test was conducted to analyze changes in the physical fitness of middle school students on an yearly basis (2019, 2020, and 2021). Post-hoc analysis was performed using Tukey’s rank test. All significance levels were set to 0.05.

## Results

### Changes in physical fitness in middle school students

The changes in the physical fitness of middle school students are presented in Table 1. BMI was significantly different between the panel data (*X*^2^ = 17.506, *p* < 0.001). As a result of post-hoc, the 2021 data had a relatively high BMI compared with that of 2019 and 2020. The 20 m shuttle run showed a significant difference among the panel data (*X*^2^ = 20.723, *p* < 0.001). As a result of the post-hoc, the 2021 data had a relatively high 20 m shuttle run compared to 2019 and 2020. There were no significant differences among the panel data in grip strength (*X*^2^ = 2.884, *p* = 0.236), sit-and-reach (*X*^2^ = 3.697, *p* = 0.158), and 50 m run (*X*^2^ = 5.670, *p* < 0.059).

### Changes in physical fitness of middle school students according to sex

Changes in the physical fitness of middle school students according to sex are presented in Table 2. The BMI of the male students was significantly different among the panel data (*X*^2^ = 6.399, *p* = 0.041). Post-hoc analysis revealed that the 2019 data had a higher BMI than the 2020 data. The BMI of the female students was significantly different among the panel data (*X*^2^ = 21.010, *p* < 0.001). Post-hoc analysis revealed that the 2021 data had a significantly higher BMI than 2019 and 2020.

**Table 2:** Changes in PAPS of middle school students according to gender by year.

		2019^a^	2020^b^	2021^c^	*X* ^2^	*p*-value	Post-hoc
BMI^#^ (kg/m^2^)	Male (*n* = 14, 941)	22.1 ± 1.1	21.7 ± 0.9	22.2 ± 1.1	6.399	0.041^*^	a, c >b
	Female (*n* = 12,841)	20.8 ± 0.6	21.1 ± 0.9	22.0 ± 1.1	23.010	<0.001^***^	a, b <c
Shuttle run^#^ (rep)	Male (*n* = 14,941)	55.3 ± 9.8	46.5 ± 14.6	38.9 ± 13.5	32.424	<0.001^***^	a >b >c
	Female (*n* = 12,841)	35.3 ± 7.8	37.9 ± 11.6	35.4 ± 11.3	0.940	0.625	–
Grip strength^#^ (kg)	Male (*n* = 14,941)	33.9 ± 4.7	30.3 ± 6.6	31.2 ± 7.7	10.314	0.006^**^	a >b, c
	Female (*n* = 12,841)	25.1 ± 2.2	26.2 ± 3.9	28.4 ± 5.1	11.777	0.003^**^	a <c
Sit and reach^#^ (cm)	Male (*n* = 14,941)	9.1 ± 2.0	11.1 ± 3.3	12.3 ± 3.4	23.159	<0.001^***^	a <b, c
	Female (*n* = 12,841)	15.5 ± 2.0	13.2 ± 3.6	13.3 ± 4.0	8.284	0.016^*^	a >b, c
50 m run^#^ (s)	Male (*n* = 14,941)	8.3 ± 0.5	8.6 ± 0.8	9.1 ± 1.2	17.864	<0.001^***^	a <b, c
	Female (*n* = 12,841)	9.7 ± 0.5	9.5 ± 0.7	9.4 ± 0.8	3.905	0.142	–

**Notes.**

mean ± SD mean and standard deviation BMI body mass index rep. repetition

* *p* < 0.05

** *p* < 0.01

*** *p* < 0.001

Kruskal–Wallis test & Tukey test using ranks. Number sign denotes the student who measured BMI, Shuttle run, Grip strength, Sit and reach, and 50 m run is the same person.

The 20 m shuttle run of male students showed a significant difference among the panel data (*X*^2^ = 32.424, *p* < 0.001). Post-hoc data showed that the 2021 data had a significantly lower 20 m shuttle run record than the 2020 data, and the 2020 data had a significantly lower 20 m shuttle run record than the 2019 data. There was no significant difference in the female 20 m shuttle run data among the panel data (*X*^2^ = 0.940, *p* = 0.625).

The grip strength of the male students was significantly different among the panel data (*X*^2^ = 10.314, *p* = 0.006). Post-hoc analysis revealed that the 2020 data had significantly lower grip strength than the 2019 data. The grip strength of the female students was significantly different among the panel data (*X*^2^ = 11.777, *p* = 0.003). Post-hoc analysis revealed that the 2021 participants had significantly lower grip strength than the 2019 participants.

The sit-and-reach test of male students showed a significant difference among the panel data (*X*^2^ = 23.159, *p* < 0.001). Post-hoc analysis revealed that the 2020 and 2021 data had significantly higher sit-and-reach rates than 2019. The sit-and-reach test of female students showed a significant difference among the panel data (*X*^2^ = 8.284, *p* = 0.016). The sit-and-reach rates in 2020 and 2021 were significantly lower than those in 2019.

The 50 m run results of male students showed a significant difference among the panel data (*X*^2^ = 17.864, *p* < 0.001). As a result of post-hoc analysis, the 2020 and 2021 data had a significantly higher 50 m run record than 2019. There was no significant difference among the panel data (*X*^2^ = 3.905, *p* = 0.141) in the female 50 m run results.

## Discussion

This study aimed to analyze the physical fitness changes of middle school students during the COVID-19 pandemic. The following results were obtained. First, middle school students showed increased BMI and decreased shuttle run performance. Second, the performance of shuttle run, grip strength, and 50 m running were decreased in male students but flexibility was increased. Third, the female student showed increased BMI and decreased flexibility; meanwhile, the grip strength was increased.

To the best of our knowledge, this is one of the first cohort studies to directly examine changes in the fitness of adolescents in South Korea during emergencies and social isolation, as seen during the COVID-19 pandemic. The current main finding is that the body composition and cardiorespiratory endurance of middle school students in Korea were negatively affected during the lockdown caused by the COVID-19 pandemic, as compared to before the lockdown (2019). Male students had significantly decreased body composition, cardiorespiratory endurance, muscle strength, and power. Female students showed a significant decrease in body composition and flexibility test results. However, flexibility among male students and muscle strength among female students improved after the pandemic. Therefore, our study provides a basis for schools to prepare policy alternatives for coping with the second wave of COVID-19 and for improving the physical fitness of the students.

The Korean Ministry of Education postponed the opening of schools in February 2020, considering the rapid increase in the number of confirmed COVID-19 cases. The situation did not improve even after the postponement; hence, school closures and online classes for students were formalized (Ministry of Education, 2020). School closures for social-behavioral adaptations, such as social distancing, quarantine, and strengthening personal hygiene, have changed the daily lives of adolescents by inculcating a sedentary lifestyle, alterations in sleep patterns, and reduced opportunities for participation in social activities. Obesity is one of the most notable physical fitness changes in adolescents owing to changes in daily life. During the lockdown, adolescents tended to have increased food intake (Stavridou et al., 2021) and sleep and screen time (Kołota & Głabska, 2021). These behaviors have been found to have a negative effect on adolescent obesity. A decrease in physical fitness along with an increase in obesity is a typical symptom experienced by adolescents, and these two variables are closely related to each other (Agurto et al., 2021). A previous longitudinal study observed fitness changes in 111 adolescents during the lockdown period in the United Kingdom, the 20 m shuttle run test (-3 shuttles) and sit-and-reach (−1.8 cm) test results were reduced compared to before the lockdown (Basterfield et al., 2022). A comparison of the changes in physical fitness of 293 adolescents during the 5-month-lockdown in Greece in 2020 and the cohort data from 2016–2017 and 2018–2019 resulted in impaired lower body fitness evaluated by jumping, sprinting, and agility tests (Tsoukos & Bogdanis, 2021). A comparison of physical fitness in 264 adolescents during the lockdown in the United States showed decreased push-ups, sit-ups, and cardiorespiratory endurance compared to before the lockdown (Wahl-Alexander & Camic, 2021). Another finding of this study was that cardiorespiratory endurance, grip strength, and 50 m run were lower among male adolescents after the lockdown in Korea. However, these reductions were not observed in female adolescents. In the United Kingdom and the United States, the same decrease in physical fitness factors was observed for both male and female students during the lockdown period (Basterfield et al., 2022; Wahl-Alexander & Camic, 2021). In Greece, the variables of physical fitness of the lower body were equally reduced; however, the flexibility and muscle strength of male students decreased in case of the upper body (Tsoukos & Bogdanis, 2021). Our study showed a different phenomenon from previous studies. The male students had a significant decrease in cardiovascular endurance, muscle strength, and power during the closure period. However, only the BMI showed a significant increase in female students, indicating that the gender of middle school students had a different effect on the physical fitness factors during the lockdown period in Korea.

Instead, female students tended to have increased muscle strength in 2020, and in 2021, muscle strength increased significantly compared to that in 2019. Therefore, it was confirmed that this trend was different from that observed in previous studies. Male students have higher levels of PA and fitness than female students, and that female students’ PA levels decrease with increasing grades (Park & Kim, 2012; Mota et al., 2011). However, the opposite result was observed after the lockdown. One possible explanation is that the decrease in the physical fitness level of male students after the lockdown may have been more significant in Korea because male students participated more frequently in competitive sports than female students. These results suggest that the change in the physical fitness of adolescents based on sex after the lockdown may differ among countries, and it is necessary to establish an education policy suitable for each country’s situation. However, it is necessary to further investigate the reason for increased women’s grip strength and decreased sit-and-reach results after the lockdown.

On the other hand, the most frequently occurring programs in school physical education classes are traditional and competitive team sports and games. Competitive team sports classes increase PA and fitness of adolescents and have positive psychological effects, such as formation of social relationships (Bailey, 2017). However, the level of PA in competitive team sports may vary among participants, depending on adolescents’ low fitness levels, obesity, and sex (Park & Kim, 2012). In particular, female Korean students were found to have a lower participation rate in PA than male students, and the content, method, and exercise skills required for the classes further limited the participation of female students in PA (Kim, Park & Choi, 2017). We also confirmed that the decrease in physical fitness of males after the lockdown in Korea was more noticeable than in female students. Based on these results, introduction of physical education classes that can overcome intrinsic (low fitness level, obesity, sex) and extrinsic (COVID-19 pandemic) factors affecting the decrease in PA, are warranted. In previous related studies, Cataldi et al. (2021) reported that an eight-week fitness program (CrossFit) during the lockdown period increased physical fitness and self-efficacy in adolescents. Lee, Noh & An (2021) investigated the effects of Tabata exercise used in online classes during lockdown. They reported that 12 weeks of Tabata improved the muscle strength, power, and balance in adolescents. Therefore, fitness programs should be added to physical education classes as an alternative method to maintain and improve the fitness level of youth in the lockdown situation caused by the COVID-19 pandemic. Moreover, it is necessary to use various advanced devices to continuously evaluate and monitor the physical fitness level of youth, and to create an environment in which physical education classes are a possibility in any situation using artificial intelligence and virtual reality. To create such an environment, governments must preemptively formulate public health policies considering the PA aspect during the pandemic (Jurak et al., 2021). However, this study analyzed PAPS data collected in 2019–2021 using a panel analysis method. Therefore, the data included the same students but was not matched to each year for individual follow-up.

## Conclusions

The WHO (2020a) recommends moderate-to-vigorous physical activity for at least 60 min each day, considering its developmental and overall impact on adolescents’ physical and mental health. Schools have the most significant impact on the health of adolescents, where vigorous PA is frequently performed. However, due to school closures during the COVID-19 pandemic, team sports with other students could no longer be performed on playgrounds and gymnasiums. During this period, the only alternative was to participate in online fitness classes that were conducted in personal spaces. Even before the COVID-19 pandemic, the participation of female students in PA in Korea was lower than males. In addition, interest in team sports was low; hence, it was necessary to change the content and methods of classes. Furthermore, many experts have predicted that a second pandemic will occur in the future. Therefore, the Ministry of Education and schools should consider fitness classes suitable for students to ensure proper maintenance of their physical fitness, in addition to traditional team sports classes. Further, it is necessary to create policies for students, especially female students, to induce their interest in classes and encourage active participation in PAs through various devices, artificial intelligence, and virtual reality devices that can measure real-time health conditions in physical education classes.

##  Supplemental Information

10.7717/peerj.14494/supp-1 Data S1 Raw data

## Additional Information and Declarations

### Data Availability

The following information was supplied regarding data availability: The raw data are available as a Supplemental File.

## References

[ref-1] Agurto HS, Alcantara-Diaz AL, Espinet-Coll E, Toro-Huamanchumo CJ (2021). Eating habits, lifestyle behaviors and stress during the COVID-19 pandemic quarantine among Peruvian adults. PeerJ.

[ref-2] Al Hourani H, Alkhatib B, Abdullah M (2021). Impact of COVID-19 lockdown on body weight, eating habits, and physical activity of Jordanian children and adolescents. Disaster Medicine and Public Health Preparedness.

[ref-3] Bailey R (2017). Sport, physical activity and educational achievement—towards an explanatory model. Sociology of Sport.

[ref-4] Basterfield L, Burn NL, Galna B, Batten H, Goffe L, Karoblyte G, Weston KL (2022). Changes in children’s physical fitness, BMI and health-related quality of life after the first 2020 COVID-19 lockdown in England: a longitudinal study. Journal of Sports Sciences.

[ref-5] Bell SL, Audrey S, Gunnell D, Cooper A, Campbell R (2019). The relationship between PA, mental wellbeing and symptoms of mental health disorder in adolescents: a cohort study. International Journal of Behavioral Nutrition and Physical Activity.

[ref-6] Cataldi S, Francavilla VC, Bonavolontà V, De Florio O, Carvutto R, De Candia M, Fischetti F (2021). Proposal for a fitness program in the school setting during the covid 19 pandemic: Effects of an 8-week crossfit program on psychophysical well-being in healthy adolescents. International Journal of Environmental Research.

[ref-7] Choi JY (2020). COVID-19 in South Korea. Postgraduate Medical Journal.

[ref-8] Fitzgerald A, Fitzgerald N, Aherne C (2012). Do peers matter? A review of peer and/or friends’ influence on PA among American adolescents. Journal of Adolescence.

[ref-9] Gill M, Chan-Golston AM, Rice LN, Cole BL, Koniak-Griffin D, Prelip ML (2016). Consistency of moderate to vigorous PA in middle school physical education. Family & Community Health.

[ref-10] Jurak G, Morrison SA, Kovač M, Leskošek B, Sember V, Strel J, Starc G (2021). A COVID-19 crisis in child physical fitness: creating a barometric tool of public health engagement for the Republic of Slovenia. Frontiers in Public Health.

[ref-11] Kim MT, Park JL, Choi JH (2017). Exploring the pleasure factors and inhibitory factors of middle school physical education classes. Korean Journal of Teacher Education.

[ref-12] Kołota A, Głąbska D (2021). COVID-19 Pandemic and remote education contributes to improved nutritional behaviors and increased screen time in a Polish population-based sample of primary school adolescents: diet and activity of youth during COVID-19 (DAY-19) study. Nutrients.

[ref-13] Lee KJ, Noh BJ, An KO (2021). Impact of synchronous online physical education classes using tabata training on adolescents during COVID-19: a randomized controlled study. International Journal of Environmental Research.

[ref-14] Lee KI, Yang TY (2021). Analysis of physical activity level and traits of male and female middle school students in daily life according to time (school days with PE class, school days without PE class, weekends) and sex. KJSP.

[ref-15] Leger L, Gadoury C (1989). Validity of the 20 m shuttle run test with 1 min stages to predict VO2max in adults. Canadian Journal of Sport Sciences.

[ref-16] Lubans DR, Smith JJ, Peralta LR. Plotnikoff RC, Okely AD, Salmon J, Morgan PJ (2016). A school-based intervention incorporating smartphone technology to improve health-related fitness among adolescents: Rationale and study protocol for theNEAT and ATLAS 2.0 cluster randomised controlled trial and dissemination study. BMJ Open.

[ref-17] Masanovic B, Gardasevic J, Marques A, Peralta M, Demetriou Y, Sturm DJ, Popovic S (2020). Trends in physical fitness among school-aged children and adolescents: a systematic review. Frontiers in Pediatrics.

[ref-18] McMahon EM, Corcoran P, O’Regan G, Keeley H, Cannon M, Carli V, Wasserman D (2017). Physical activity in European adolescents and associations with anxiety, depression and well-being. European Child & Adolescent Psychiatry.

[ref-19] Ministry of Education (2009). Student Health and Physical Fitness Assessment System (PAPS) measurement manual. https://www.moe.go.kr/boardCnts/viewRenew.do?boardID=316boardSeq=14508lev=0searchType=nullstatusYN=Cpage=1s=moem=0302opType=N.

[ref-20] Ministry of Education (2020). Current Status of COVID-19 in the Field Education. https://www.moe.go.kr/sub/infoRenew.do?m=031301page=031301s=moe.

[ref-21] Ministry of Education (2022a). Basic plan for school sports revitalization. https://www.moe.go.kr/boardCnts/viewRenew.do?boardID=294boardSeq=90895lev=0searchType=nullstatusYN=Wpage=1s=moem=020402opType=N.

[ref-22] Ministry of Education (2022b). ‘COVID-19 response in the field of education’ published white paper. https://www.moe.go.kr/boardCnts/viewRenew.do?boardID=294lev=0statusYN=Ws=moem=020402opType=NboardSeq=91497.

[ref-23] Mittal VA, Firth J, Kimhy D (2020). Combating the dangers of sedentary activity on child and adolescent mental health during the time of COVID-19. Journal of the American Academy of Child and Adolescent Psychiatry.

[ref-24] Mota J, Santos R, Pereira M, Teixeira L, Santos MP (2011). Perceived neighbourhood environmental characteristics and PA according to socioeconomic status in adolescent girls. Annals of Human Biology.

[ref-25] Park JG, Kim KW (2012). Physical activity level according to gender, grades, body mass index, and personality traits in adolescents. The Asian Journal of Kinesiology.

[ref-26] Rangul V, Bauman A, Holmen TL, Midthjell K (2012). Is PA maintenance from adolescence to young adulthood associated with reduced CVD risk factors, improved mental health and satisfaction with life: the HUNT Study, Norway. International Journal of Behavioral Nutrition and Physical Activity.

[ref-27] Rhodes RE, Janssen I, Bredin SS, Warburton DE, Bauman A (2017). Physical activity: health impact, prevalence, correlates and interventions. Psychology & Health.

[ref-28] Stavridou A, Kapsali E, Panagouli E, Thirios A, Polychronis K, Bacopoulou F, Tsitsika A (2021). Obesity in children and adolescents during COVID-19 pandemic. Children.

[ref-29] Tsoukos A, Bogdanis GC (2021). The effects of a five-month lockdown due to COVID-19 on physical fitness parameters in adolescent students: a comparison between cohorts. International Journal of Environmental Research.

[ref-30] Wahl-Alexander Z, Camic CL (2021). Impact of COVID-19 on school-aged male and female health-related fitness markers. Pediatric Exercise Science.

[ref-31] World Health Organization (WHO) (2020a). Physical activity. https://www.who.int/news-room/fact-sheets/detail/physical-activity.

[ref-32] World Health Organization (WHO) (2020b). WHO characterizes COVID-19 as a pandemic. https://www.who.int/emergencies/diseases/novel-coronavirus-2019/events-as-they-happen.

[ref-33] Yu YW, Song YK, Yu MS, Jeon YK (2018). Association between physical activity and academic problems due to smartphone. JKSSPE.

